# Effects of Astragaloside IV on Hearing, Inflammatory Factors, and Intestinal Flora in Mice Exposed to Noise

**DOI:** 10.3390/metabo14020122

**Published:** 2024-02-11

**Authors:** Junyi Li, Jian Yang, Yun Xia, Junyi Wang, Yuan Xia

**Affiliations:** School of Public Health, Guangdong Pharmaceutical University, Guangzhou 510310, China; 2112141084@gdpu.edu.cn (J.L.); 2112241062@gdpu.edu.cn (J.Y.); xy@gdpu.edu.cn (Y.X.)

**Keywords:** astragaloside IV, noise, hearing loss, intestinal flora, auditory brainstem response, inflammatory factors

## Abstract

Long-term exposure to noise can cause irreversible hearing loss. Considering that there is no effective drug treatment, it is important to seek preventive treatment for noise-induced hearing loss (NIHL). Although astragaloside IV (AS-IV) protects against NIHL by reducing serum inflammatory factors, there is scarce information on the regulation of inflammatory factors by AS-IV to prevent NIHL. We investigated the hearing thresholds and relationship between the serum levels of inflammatory cytokines and intestinal microbiota of c57bl/6j mice exposed to noise (103 dB SPL 4 h·d^−1^) for 7 days, treated with or without AS-IV. Our results revealed a lower hearing threshold and lower serum levels of TNF-α, TNF-γ, IL-6, IL-1β, and IFN-γ in the mice treated with AS-IV. Additionally, AS-IV increased the abundance levels of the phylum *Firmicutes*, class *Bacillus*, order *Lactobacillus*, and family *Lactobacillus* (*p* < 0.05), and decreased those of the phylum *Bacteroidetes* and order *Bacteroidales* (*p* < 0.05). *Lactobacillus* and *Bacilli* negatively correlated with TNF-α, TNF-γ, and IL-1β; *Erysipelotrichaceae* negatively correlated with INF-γ; and *Clostridiales* positively correlated with IL-1β. In conclusion, AS-IV reduces the elevation of hearing thresholds in mice, preventing hearing loss in mice exposed to noise, and under the intervention of AS-IV, changes in the levels of inflammatory factors correlate with intestinal flora. We suggest that AS-IV improves intestinal flora and reduces inflammation levels in c57bl/6j mice exposed to noise.

## 1. Introduction

Approximately 5% of the global population is affected by noise-induced hearing loss (NIHL), which imposes an enormous economic and health burden on individuals and society [1]. Furthermore, occupational noise-induced deafness is the second most common occupational disease and affects approximately 16% of workers [2]. According to a systematic review, the noise-induced permanent threshold shift after 10 years of exposure at *L*_ex,8h_ = 100 dB of 3–6 kHz is 27 dB [3]. A recent study on manufacturing workers has shown that the mean prevalence of high-frequency NIHL is 41.64% [4]. Although current research on NIHL drug therapy includes anti-inflammatory (such as dexamethasone [5], methylprednisolone [6], and curcumin [7]), antioxidant (such as resveratrol [8] and salicylate Trolox [9]), and antiapoptotic drugs (such as all-trans retinoic acid [10] and AM-111 [11]), there are no specific drugs for NIHL [1]. In clinical practice, hearing aids [12] and cochlear implants [13] are generally supportive treatments for permanent hearing loss, but the patients can neither fundamentally repair damaged or missing hair cells (HCs), nor they can achieve complete reconstruction of hearing function. Because hearing cells are considered terminal cells, they cannot regenerate once severely damaged [14]. Therefore, it is important to clarify the pathological mechanism of NIHL and seek effective preventive drugs for NIHL. In this study, we used the noise intensity of production work (*L*_ex,8h_ ≤ 100 dB(A)) [3] with broadband noise in which harm to the auditory system may be less than an octave band to construct a NIHL model [15]; the noise should cause NIHL or pathological manifestations of outer hair cells but not cause too serious damage to avoid the excessive use of high doses of AS-IV.

Inflammatory infiltration and HC deficiency are important contributors to the pathogenesis of NIHL [16,17]. In particular, at an early stage of the development of NIHL, the levels of IL-6, TNF-α, IL-1β, and other inflammatory factors are increased [18]. One study showed that the average loss of outer hair cells (OHCs) in mice exposed to 120 dB SPL for 1 h was 29.3% [19], and another study showed that the highest loss of OHCs was over 60% in mice exposed to noise at 105 dB SPL for 2 h [20]. Noise stimulates HCs, making them active, producing an increase in mitochondria [21], and leading to superoxide production and reactive oxygen species (ROS) production [22]. The destruction of cochlear hair cells can lead to permanent noise-induced hearing loss [23,24,25]. Therefore, in this study, we considered that hearing loss had occurred or was about to occur when the pathological changes of outer hair cell loss and shedding occurred, and then ABR was used to judge the hearing state of the mice at that time [26,27].

Recent studies have shown that oral administration of AS-IV at a dose of 200 mg/kg·d^−1^ may reduce the elevation of hearing thresholds by decreasing the expression of ROS and active-caspase-3 in a noise-exposed guinea pig model [28]. Furthermore, AS-IV has significant pharmacological effects, such as an anti-inflammatory effect on cartilage degeneration in patients with osteoarthritis [29], an antioxidant effect that inhibits the NLRP3/caspase-1 axis to inhibit NLRP3 inflammasome-mediated pyroptosis [30], and an immunomodulatory effect by regulating the T-cell receptor signaling pathway and Th17 cell differentiation [31]. AS-IV can alleviate *E. coli*-induced peritonitis by regulating neutrophil migration [32]. However, the specific mechanism by which AS-IV prevents noise-induced deafness is still unclear.

Although AS-IV (molecular formula, C_41_H_68_O_14_) is a saponin with poor absorption, and the absolute bioavailability of AS-IV in rats with oral administration is 3.66% [33], the abundance of gut microorganisms and fecal metabolites is altered by AS-IV, which may contribute to their antifibrotic and cardioprotective effects [34]. One study showed that 105 dB SPL noise exposure affected gut microbiota and metabolic disorders in rats [35]. Long-term low-intensity noise exposure can increase the abundance of *Firmicutes* and diminish that of *Bacteroidetes* in intestinal flora, and it can induce cognitive decline in mice [36]. Moreover, chronic noise exposure significantly reduces the abundance of *Rikenellaceae*, *Ruminococcaceae*, *Anaerobia*, *Lachnospira*, and *Odoribacter* in the microbial community and significantly increases the levels of IL-6, NF-κB, iNOS, and NGAL in the mouse intestine [37].

According to a previous study, AS-IV might reduce noise-induced increases in hearing threshold by reducing inflammatory factors [38,39], and AS-IV could improve disease prognosis by ameliorating intestinal flora with optimizing intestinal metabolites [40]. However, the correlation between inflammatory factors and intestinal flora of mice in which NIHL is protected by AS-IV remains poorly understood [39,41]. Therefore, the present study aimed to initially explore the mechanism by which AS-IV reduces NIHL damage through synergistic alterations in gut microbiota and inflammation.

## 2. Materials and Methods

### 2.1. Animals Groups

Eighteen 6-week-old specific-pathogen-free male c57bl/6j mice, weighing approximately 14–16 g, were purchased from the GuangDong Medical Laboratory Animal Center (License No. SCXK 2022-0002, GDMLAC, Foshan, China). The mice were housed in the Laboratory Animal Center, Guangdong Pharmaceutical University under the following conditions: temperature of 20 ± 0.5 °C, relative humidity of 55% ± 5%, and a light/dark period of 12 h. All experimental procedures were approved by the Animal Ethics Committee of Guangdong Pharmaceutical University (No. gdpulacspf2022124). The mice were randomly divided into three groups, namely the control group, the noise-exposed group, and the AS-IV group, with six mice in each group. AS-IV (>98%, Macklin, Shanghai, China) with 0.05% sodium carboxymethylcellulose was prepared as suspension and administered by gavage at 100 mg/kg body weight once a day until the end of experimental exposure [38,39]. The 6-week-old mice were purchased and subjected to a week of environmental acclimation, with the first ABR and the beginning of exposure to noise and AS-IV at 7 weeks of age, the end of noise exposure at 8 weeks of age, and the last ABR at 10 weeks of age.

### 2.2. Noise Exposure and Procedures

The mice were placed in a small metal cage (7 × 7 × 10 cm) in a self-built noise exposure box (50 × 50 × 40 cm). The noise was broadband noise with a frequency of 20 Hz–20 kHz [42]. A broad-spectrum loudspeaker (FEI L0 YD3-2001; Guangzhou, China) was positioned 10 cm above the metal cage. The loudspeaker received noise transmitted from a computer and through a power amplifier for cyclic playback. The noise-exposed group was exposed to 103 dB SPL noise for 7 days in a row for 4 h a day (L_EX,8h_ = 100.0 dB) [3]. The control mice were kept in a quiet room with less than 40 dB SPL, while other conditions were the same as those in the noise-exposed group.

### 2.3. Auditory Brainstem Response (ABR) Audiometry

Aiming to determine the hearing level, the hearing thresholds of the c57 mice were measured before noise exposure and 14 days after the end of exposure. The c57 mice were anesthetized by intraperitoneal injection of 40 mg/kg of 1% sodium pentobarbital. ABR audiometry was conducted using the Neuro-Audio device from Neurosoft (Ivanovo, Russia) and was recorded using the supporting system (NEURO-AUDIO.NET). The temperature of the c57 mice was maintained using a heating pad. Left and right reference electrodes were inserted subcutaneously behind each tested ear, and the recording electrodes were inserted into the vertex. The tested ears of the mice were presented with click and tone burst stimuli (4, 8, and 16 kHz) through a pair of in-ear headphones. The signal started at 100 dB; the stride was 10 dB initially and 5 dB when approaching the threshold. The results were set as 512 repetitions of the stimulus superposition waveform, the stimulus frequency was 20 times per second, the low-frequency filter was 100 Hz, and the high-frequency filter was 2000 Hz. The minimum sound stimulus intensity that could cause wave III and a reproducible waveform was defined as the response threshold to evaluate the hearing impairment of mice in each group [43,44,45].

### 2.4. Observation of Cochlear Hair Cells

After the mice were sacrificed, the tissues around the acoustic bulla of the temporal bone were removed, and the cochlea was isolated, fixed in 4% paraformaldehyde, and decalcified with 0.5 M EDTA to suitable hardness. The basilar membrane was dissected under a stereoscopic microscope (SZ760; Cnoptec, Chongqing, China). The isolated basilar membrane was permeabilized with 0.1% Triton X-100 (Amresco, Washington, DC, USA) for 30 min, stained with bovine serum albumin to prepare Actin-Tracker Green (Beyotime, Shanghai, China) for 70 min, and washed with phosphate-buffered saline two to three times. Pictures were taken under a benchtop microscope (EVOS^TM^ M5000; Thermo Fisher Scientific, Waltham, MA, USA).

### 2.5. Analysis of Inflammatory Indicators

Blood was collected using the orbital blood collection method [46], and mouse serum was obtained after centrifugation. An ELISA kit (MEIMIAN, Yancheng, China) was used to detect TNF-α, TNF-γ, IL-6, IL-1β, and IFN-γ content in mouse serum. We created a concentration absorbance standard curve based on the standard sample and calculated the concentration of the inflammatory substances in the sample according to the standard curve.

### 2.6. Analysis of the Microbiota

Mouse fecal samples were obtained by stimulating the tail and stored in sterile centrifuge tubes at −80 °C, which were used to identify the 16S rRNA gene sequence. Then, the DNA of the corresponding genome was extracted following the instructions of the kit manufacturer. Integrity, purity, and concentration of DNA were determined by 1% agarose gel electrophoresis and an ultramicrophotometer. Polymerase chain reaction was used to amplify genomic DNA. A database was constructed and sequenced using the previous methods [47]. Then, based on valid sequencing data, Operational Taxonomic Uni clustering and species analysis were conducted, and annotation was performed to obtain species information and distribution of the microbiota in each group. The microbiota histogram was used to analyze the composition of the microbiota at the phylum, class, order, family, and other levels.

### 2.7. Statistical Analysis

All data were analyzed using GraphPad Prism version 9.0.0 for Windows (GraphPad Software, La Jolla, CA, USA) and expressed as the mean ± standard deviation. Analysis of single-factor differences between groups was achieved using one-way analysis of variance, Brown–Forsythe and Welch ANOVA tests. Multiple pairwise comparisons were based on the least-significant-difference method. Correlation analysis was conducted using the *Spearman* test. The significance level was set at α = 0.05.

## 3. Results

### 3.1. AS-IV Improves Hearing in Noise-Exposed Mice

We assessed the level of hearing in the mice using ABR. The hearing thresholds of the mice increased after noise exposure (click: 21.3 dB vs. 42.5 dB, *p* < 0.0001; 4 kHz: 26.9 dB vs. 54.4 dB, *p* < 0.0001; 8 kHz: 23.8 dB vs. 37.7 dB, *p* < 0.0001; and 16 kHz: 27.1 dB vs. 43.3 dB, *p* = 0.0002, indicating increases of 21.2 dB, 27.5 dB, 13.9 dB, and 16.2 dB, respectively). After taking AS-IV, the hearing threshold of the mice was lower than that of the noise-exposed group at click, 4 kHz, 8 kHz, and 16 kHz.

The hearing thresholds of the mice in the AS-IV group at click, 4 kHz, 8 kHz, and 16 kHz were 13.1 dB, 16.4 dB, 11.7 dB, and 10.8 dB, respectively, which were lower than those in the noise-exposed group (*p* < 0.05) (Table 1).

### 3.2. AS-IV Reduces the Loss of OHCs in Noise-Exposed Mice

**Figure 1:**
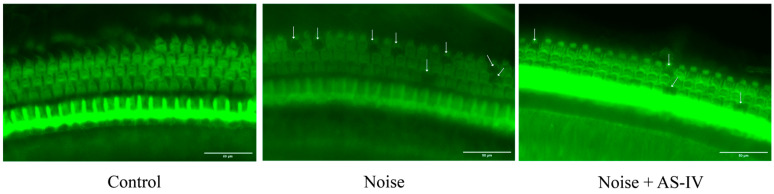
Fluorescence image of middle turns of mouse hair cells (scale bar, 50 μm; the arrows point to the missing outer hair cells).

The OHCs of the control group mice were arranged neatly without any cell loss. After 14 days of noise exposure, the cochlear OHCs of the noise-exposed group showed significant deformation; the damage to the OHCs (Figure 1) was more serious in the noise group, whereas the damage to OHCs giving AS-IV was small or absent. The OHC loss in the mice from the AS-IV group was lower than that in the noise-exposed group. Combined with the ABR results, our findings indicate that AS-IV can alleviate hearing loss.

### 3.3. AS-IV Reduces the Levels of Inflammatory Factors in Noise-Exposed Mice

The levels of TNF-α, TNF-γ, IL-6, IL-1β, and IFN- γ were significantly increased in the noise-exposed group compared with the control group (by 158.7%, 78.5%, 45.8%, 42.1%, and 38.8%, respectively; *p* < 0.05). After treatment with AS-IV, the levels of TNF-α, TNF-γ, and IL-1β were significantly decreased compared with those in the noise-exposed group (*p* < 0.05) (Table 2).

### 3.4. AS-IV Alleviates the Disorder of Intestinal Flora in Noise-Exposed Mice

**Figure 2:**
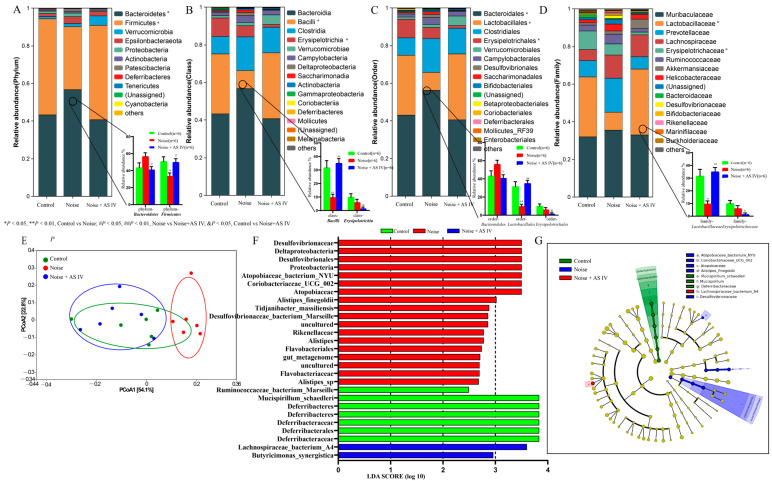
Effect of AS-IV on intestinal flora in noise-exposed mice. (**A**–**D**) The distribution of the main dominant microbiota under phyla, class, order, and family in each group. (**E**) Principal coordinate analysis of the three groups based on Bray–Curtis algorithm. (**F**) Only the taxa meeting an LDA threshold > 2 are visualized. (**G**) LEfSe cladogram shows the most differentially abundant taxa for the three groups.

The dominant microbiota in the mice were *Firmicutes* (50.1%), *Bacteroidetes* (40.7%), *Verrucomicrobia* (5.1%), *Epsilonbacteraeota* (2.5%), and *Proteobacteria* (1.3%). Noise exposure resulted in decreases in the abundance of phylum *Firmicutes*, phylum *Bacteroidetes*, class *Bacilli*, order *Lactobacillales*, order *Bacteroidales*, and family *Lactobacillaceae* (*p* < 0.05). However, administration of AS-IV increased the abundance levels of phylum *Firmicutes*, class *Bacilli*, order *Lactobacillales*, and family *Lactobacillaceae* in the intestine of the noise-exposed mice (*p* < 0.05) (Figure 2A–D).

Using principal coordinate analysis (PCoA) to evaluate the overall differences in the gut microbiota structure of the mice caused by noise exposure, principal coordinates 1 and 2 explained 54.1% and 22.8% of the Bray–Curtis differences, respectively. The difference in the microbiota structure was statistically significant between the noise-exposed group and control group and between the noise group and the noise + AS-IV group. The difference between the control group and the noise + AS-IV group was not significant (Figure 2E).

The results of linear discriminant analysis (LDA > 2) showed that in the control group, the main enriched bacterial class was *Deferribacteria*, and the main bacterial species was *Mucispirillum_Schaedeleri.* The main enriched bacterial phyla in the noise group were *Firmicutes*, *Bacteroidetes*, *Proteobacteria*, and *Actinobacteria.* The main enriched bacterial species in the noise group were *Ruminococcaceae_bacterium_Marseille*, *Alistipes_sp*, and *gut_metagenome*. The species *Lachnospiraceae_Bacterium_A4* of the phylum *Bacteroidetes and* the species *Butyricimonas_synergistica* of the phylum *Firmicutes* were enriched in the noise + AS-IV group (Figure 2F).

### 3.5. Correlation Analysis between Gut Microbiota and Inflammatory Factors

**Figure 3:**
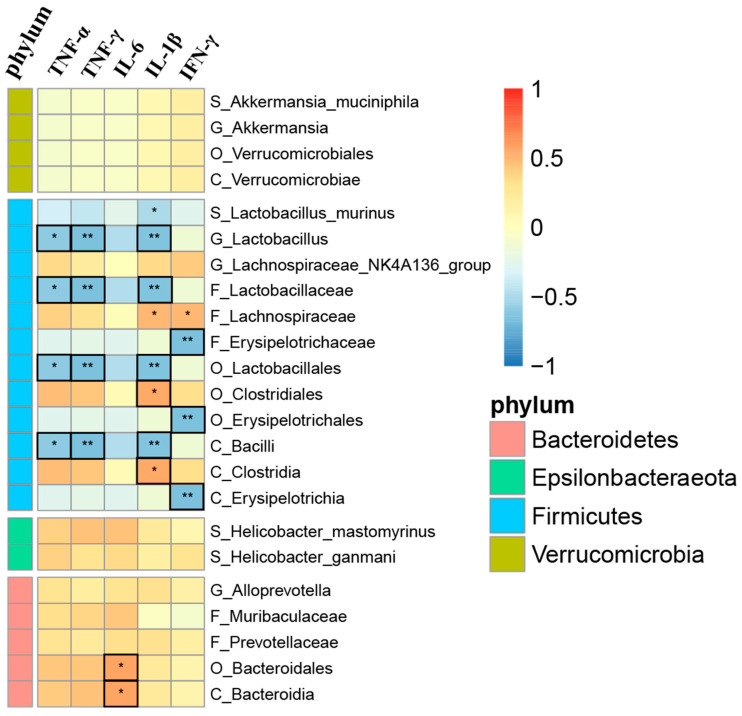
Correlation between main gut microbiota and inflammatory factors. Black boxes indicate strong correlation (|r| > 0.50, *p* < 0.05); * means significance at *p* < 0.05; and ** means significance at *p* < 0.01.

We generated a correlation matrix through the Spearman correlation coefficient and selected the top five bacterial communities in class, order, family, genus, and species for correlation analysis with mouse serum inflammatory indicators. Among them, the order *Lactobacillales*, family *Lactobacillaceae*, genus *Lactobacillus*, and class *Bacilli* were associated with serum TNF-α, TNF-γ, and IL-1β. There was a strong negative correlation of *Erysipelotrichia*, *Erysipelotrichales*, and *Erysipelotrichaceae* with IFN-γ and a strong positive correlation of *Clostridia* and *Clostridiales* with IL-1β (Figure 3).

## 4. Discussion

We showed that AS-IV had beneficial effects on hearing, which has rarely been studied before. Namely, not only did AS-IV reduce the inflammatory levels in the noise-exposed mice and regulate their intestinal flora, but it also reduced the noise-induced loss of outer hair cells and slowed noise-induced hearing loss.

We established an NIHL mouse model by exposing the mice to 103 dB SPL of white noise for 4 h per day for 7 consecutive days and testing for relevant indicators. Furthermore, we examined whether oral administration of AS-IV altered the hearing level of the mice exposed to noise. Before noise exposure, the baseline hearing levels were determined through ABR. The average hearing thresholds of the mice at click, 4 kHz, 8 kHz, and 16 kHz were 19.7 ± 6.0 dB, 25.6 ± 6.8 dB, 16.2 ± 3.6 dB, and 16.7 ± 5.0 dB, respectively, consistent with the existing studies [48]. After noise exposure, there was a significant increase in the hearing threshold (*p* < 0.05). To explore the effect of noise exposure on mice HCs, we detected the cochlear HCs of the mice using immunofluorescence with phalloidin, which can bind specifically to the cytoskeleton of HCs. The results showed that the noise of this intensity caused the loss of OHCs, while there was less damage to OHCs following AS-IV treatment (Figure 1). This finding indicates that AS-IV prevents noise-induced OHC outright loss and plays a protective role in hearing [49,50].

In addition, we analyzed whether oral administration of AS-IV altered the serum levels of inflammatory factors in the mice exposed to high-intensity noise. The results showed that TNF-α, TNF-γ, IL-6, sIL-1β, and IFN-γ content in the serum increased (*p* < 0.05), consistent with previous research [51,52,53,54,55]. The increase in the levels of TNF-α and IL-1β reflects the activation of monocytes and macrophages, which transform into macrophages and migrate below the basilar membrane, promoting inflammation and damage in the cochlear HCs [56]. Moreover, strong noise enhances the increased production of Ca^2+^ through cytokine TNF-α-mediated TRPV1 induction, while Ca^2+^ promotes the inflammation and apoptosis of inner ear tissue and cells [55]. Moon S K et al. [57] found that IFN-γ increased the sensitivity of HCs to lower-concentration Ca^2+^ cytotoxicity through the JAK1/1-STAT2 signaling pathway, which further promoted apoptosis of HCs. Marshall et al. [58] studied TNF-α as a proinflammatory cytokine and its receptor, TNF-α receptor, and showed that they play a key role in cell death mechanisms, including necrotic apoptosis and apoptosis. In addition, our study showed that the increased expression levels of the proinflammatory cytokines IL-1β and IL-6 in the mice exposed to noise were attenuated after administration of AS-IV. IL-6 is a key inflammatory marker in many mouse models, including the age-related hearing loss mouse model [59] and the COPD (chronic obstructive pulmonary disease)-like inflammatory mouse model [60]. IL-6 also induces excessive production of vascular endothelial growth factor, resulting in enhanced angiogenesis and increased vascular permeability [61], which is associated with progressive hearing loss in Meniere’s disease [62]. A study of pediatric otitis patients found a large amount of inflammatory factor IL-1β in the ear effusion [63]. IL-1β is a proinflammatory cytokine mainly produced by monocytes and macrophages, and it promotes cell apoptosis [64]. Similar to this research, Sai et al. [65] found that IL-1β content in HCs was significantly increased when the miniature pigs were exposed to 120 dB. In addition, Zhang et al.’s [66] research on Meniere’s disease indicated that the significantly increased content of IL-1β was due to serum/glucocorticoid-inducible kinase-1 depletion, which led to the damage to ear OHCs and the vestibular nerve. However, AS-IV decreased the protein levels of TNF-α and IL-6 in adipocytes through the miR-21/PTEN/PI3K/AKT signaling pathway [67]. Other studies have shown that AS-IV can interact with the gut microbiota and be decomposed and used by the gut microbiota, inducing the production of various beneficial short-chain fatty acids, which can reduce the degree of systemic inflammation [39,68].

The dominant groups in the mice intestinal flora included *Firmicutes* (50.1%) and *Bacteroidetes* (40.7%). We showed that the proportions of *Firmicutes*, *Bacilli*, and *Lactobacillales* decreased (*p* < 0.05) in mice after noise exposure, while the proportions of *Firmicutes, Bacilli, and Lactobacillales* increased (*p* < 0.05) and the proportions of *Bacteroidetes and Bacteroidales* decreased (*p* < 0.05) in the noise-exposed mice treated with AS-IV. Therefore, the preventive effect of AS-IV was partially achieved by reversing the structure of the intestinal microbiota. The gut microbiota of healthy humans mainly consists of *Firmicutes* (49%) and *Bacteroidetes* (23%), where *Firmicutes* mainly include *Clostridia*, *Bacilli*, and *Mollicutes*, and *Bacteroidetes* mainly include *Bacteroidales* and *Flavobacteriaceae* [69]. It has been shown that dietary factors have a significant impact on *Firmicutes*, and *Firmicutes* can also produce butyrate salts, which reduce the level of inflammation in the body [70]. *Bacilli* contain *Bacillales* and *Lactobacillales*, and *Lactobacillales* were shown to play an important role in the microbial community in this study. Our experiment showed that the relative abundance of *Lactobacillales* decreased after noise exposure. *Lactobacillales* are probiotics that mediate the metabolism of tryptophan into norharman and inhibit M1-type macrophages [71]. Qiulan et al. [72] found an increased abundance of AI-IV and increased content of butyric acid and valeric acid in the intestine to improve slow transit constipation. Our research results showed that *Lactobacillales* returned to the level of the control group without any abnormal increase when taking AS-IV, and did not cause microbial community disorders [73]. *Bacteroidales* is one of the most abundant members of the mammalian gut microbiota and an important microbiota for synthesizing sphingolipids in the intestine [74]. Moreover, sphingolipids mediate metabolic and immune signaling events related to chronic inflammatory diseases such as autoimmune and chronic enteritis [75]. *Bacteroidales* mainly colonize the colon and use dietary oligosaccharides such as arabinose and high galacturonic acid [76]. Their survival and reproduction in the intestine depend on the monosaccharides they use and their interactions with butyrate salts [77]. The changes in *Bacteroidales* abundance in this study may be related to the alteration in intestinal butyrate content caused by AS-IV administration [72].

Furthermore, to explore the specific mechanisms, *Spearman* correlation coefficients were used to investigate whether there was a correlation between inflammatory factors in the serum of the noise-exposed mice and their gut microbiota. Our results indicated that *Lactobacillus* negatively correlated with the mouse serum inflammatory factors TNF-α, TNF-γ, and IL-1β (*R* = −0.59, *p* = 0.012; *R* = −0.66, *p* = 0.004; and *R* = −0.63, *p* = 0.006). TNF-α and TNF-γ are proinflammatory cytokines secreted by macrophages, monocytes, and T lymphocytes, which have the effects of promoting cell apoptosis and inflammation through the activation of transcription factor NF-κB [78]. *Lactobacillales* are probiotics that can inhibit the release of proinflammatory cytokines (such as TNF-α) and promote the release of anti-inflammatory factors such as IL-10 [79]. TNF-α positively correlates with sensorineural hearing loss [59]. A retrospective cohort study suggested that inhibiting treatment with AS-IV can improve or even restore patient hearing [80]. This is because TNF-α blockers can reduce cellular inflammatory response, reduce cochlear HC apoptosis, and alleviate hearing loss [81]. After the use of AS-IV in this study, the abundance of *Lactobacillales* increased, and inflammatory mediators decreased, providing a protective effect on the body. It has been speculated that AS-IV may exert systemic anti-inflammatory and antioxidant effects by increasing intestinal volume, thereby protecting hearing [82]. Our results also showed a positive correlation between *Bacteroidales* and serum IL-6 (*R* = 0.57, *p* = 0.015), consistent with some previous studies [83]. On the one hand, *Bacteroidales* cause intestinal T cells and macrophages to produce IL-6 through the MyD88 pathway [84], inducing systemic and local inflammation [85,86]. On the other hand, *Bacteroidales* recruit colonic epithelial lymphocytes to maintain intestinal epithelial barrier function [84]. Our findings suggest that noise can cause an increase in harmful gut microbiota. The use of AS-IV in this experiment significantly reduced the increasing trend of *Bacteroidetes*. It could be speculated that AS-IV may reduce the level of IL-6 in the body by reducing the abundance of *Bacteroidetes*, thereby protecting the hearing system from damage.

## 5. Conclusions

Our study showed that AS-IV intervention in the intestinal microbiota of the noise-exposed mice correlated with inflammatory factors, showing a positive correlation of *Bacteroidales* with IL-6 (*R* = 0.57, *p* = 0.015) and a negative correlation with TNF-α and IL-1β (*R* = −0.59, *p* = 0.012, and *R* = −0.63, *p* = 0.006). We confirmed that AS-IV protected the noise-exposed mice against hearing loss (*p* < 0.05) by reducing the inflammatory indicators in the circulatory system, which was correlated with gut microbiota. Therefore, we hypothesize that AS-IV relies on changes in gut microbiota abundance to reduce inflammation levels in the body and protect the hearing system from noise damage, which provides a reference for the application of AS-IV in the prevention and treatment of some noise-induced diseases.

## Figures and Tables

**Table 1:** Hearing threshold results of mice in the control, noise-exposed, and noise + AS-IV groups before and after noise exposure ($\overset{-}{x}$ ± *s*, dB SPL).

Group	No. of Ears	Pre-Exposure				Post-Exposure			
		Click	4 kHz	8 kHz	16 kHz	Click	4 kHz	8 kHz	16 kHz
Control	12	25.3 ± 5.5	31.1 ± 2.9	20.8 ± 12.5	22.1 ± 8.5	21.3 ± 5.7	26.9 ± 6.8	23.8 ± 7.9	27.1 ± 8.7
Noise	12	19.7 ± 6.0	25.6 ± 6.8	16.2 ± 3.6	16.7 ± 5.0	42.5 ± 6.1 ^a^	54.4 ± 11.6 ^a^	37.7 ± 7.1 ^a^	43.3 ± 9.1 ^a^
Noise + AS-IV	12	23.3 ± 5.5	27.4 ± 6.7	14.9 ± 6.8	18.5 ± 5.2	29.4 ± 10.1 ^bc^	38.1 ± 10.7 ^bc^	26.0 ± 8.3 ^b^	32.5 ± 10.6 ^b^
*F*		3.03	2.82	1.63	2.15	24.18	23.26	11.07	9.11
*p* value		0.062	0.074	0.212	0.133	<0.001	<0.001	<0.001	<0.001

^a^ *p* < 0.05, control vs. noise. ^b^ *p* < 0.05, noise + AS-IV vs. noise. ^c^ *p* < 0.05, control vs. noise + AS-IV.

**Table 2:** TNF-α, TNF-γ, IL-6, IL-1β, and IFN-γ content of each group of mice ($\overset{-}{x}$ ± *s*, pg/mL).

Group	No. of Mice	TNF-α	TNF-γ	IL-6	IL-1β	IFN-γ
Control	6	152.2 ± 15.7	33.1 ± 10.8	77.3 ± 15.7	71.9 ± 6.8	585.3 ± 50.0
Noise	6	393.8 ± 51.3 ^a^	59.1 ± 8.3 ^a^	112.7 ± 13.6 ^a^	102.2 ± 13.0 ^a^	812.4 ± 93.3 ^a^
Noise + AS-IV	6	277.8 ± 86.7 ^bc^	46.6 ± 6.9 ^bc^	96.4 ± 17.4	83.5 ± 6.2 ^bc^	820.4 ± 22.8 ^c^
*F*		25.31	13.02	7.71	16.59	27.38
*p* value		<0.001	<0.001	0.005	<0.001	<0.001

^a^ *p* < 0.05, control vs. noise. ^b^ *p* < 0.05, noise + AS-IV vs. noise. ^c^ *p* < 0.05, control vs. noise + AS-IV.

## Data Availability

The data presented in this study are available in the article.
